# Differential Feedback Regulation of Δ^4^-3-Oxosteroid 5β-Reductase Expression by Bile Acids

**DOI:** 10.1371/journal.pone.0170960

**Published:** 2017-01-26

**Authors:** Leila Valanejad, Christina Nadolny, Stephanie Shiffka, Yuan Chen, Sangmin You, Ruitang Deng

**Affiliations:** Department of Biomedical and Pharmaceutical Sciences, Center for Pharmacogenomics and Molecular Therapy, College of Pharmacy, University of Rhode Island, Kingston, Rhode Island, United States of America; Texas A&M University, UNITED STATES

## Abstract

Δ^4^-3-oxosteroid 5β-reductase is member D1 of the aldo-keto reductase family 1 (AKR1D1), which catalyzes 5β-reduction of molecules with a 3-oxo-4-ene structure. Bile acid intermediates and most of the steroid hormones carry the 3-oxo-4-ene structure. Therefore, AKR1D1 plays critical roles in both bile acid synthesis and steroid hormone metabolism. Currently our understanding on transcriptional regulation of AKR1D1 under physiological and pathological conditions is very limited. In this study, we investigated the regulatory effects of primary bile acids, chenodeoxycholic acid (CDCA) and cholic acid (CA), on AKR1D1 expression. The expression levels of AKR1D1 mRNA and protein *in vitro* and *in vivo* following bile acid treatments were determined by real-time PCR and Western blotting. We found that CDCA markedly repressed AKR1D1 expression *in vitro* in human hepatoma HepG2 cells and *in vivo* in mice. On the contrary, CA significantly upregulated AKR1D1 expression in HepG2 cells and in mice. Further mechanistic investigations revealed that the farnesoid x receptor (FXR) signaling pathway was not involved in regulating AKR1D1 by bile acids. Instead, CDCA and CA regulated AKR1D1 through the mitogen-activated protein kinases/c-Jun N-terminal kinases (MAPK/JNK) signaling pathway. Inhibition of the MAPK/JNK pathway effectively abolished CDCA and CA-mediated regulation of AKR1D1. It was thus determined that AKR1D1 expression was regulated by CDCA and CA through modulating the MAPK/JNK signaling pathway. In conclusion, AKR1D1 expression was differentially regulated by primary bile acids through negative and positive feedback mechanisms. The findings indicated that both bile acid concentrations and compositions play important roles in regulating AKR1D1 expression, and consequently bile acid synthesis and steroid hormone metabolism.

## Introduction

Bile acids are the ultimate metabolites of cholesterol. Studies in the past decade uncovered a broad spectrum of functions associated with bile acids as hormone-like signaling molecules through various nuclear receptors, notably farnesoid x receptor (FXR) and G protein-coupled bile acid receptor 5 (TGR5) [1–3]. Both FXR and TGR5 signaling pathways play critical roles in regulating bile acids, cholesterol, lipids and glucose homeostasis [4–7]. Bile acid homeostasis is maintained through tightly regulated bile acid synthesis and enterohepatic circulation. Cholesterol 7α-hydroxylase (CYP7A1) is the rate limiting enzyme in classical bile acid synthesis while sterol 12α-hydroxylase (CYP8B1) is the determinant enzyme for the production of CA [8–11]. Canalicular secretion of bile acids through bile salt export pump (BSEP) is the rate-limiting step in the enterohepatic circulation of bile acids [12, 13]. Activation of FXR by bile acids represses CYP7A1 while induces BSEP expression to maintain hepatic bile acid homeostasis [14, 15].

Aldo-keto reductase family 1, member D1 (AKR1D1) is a Δ^4^-3-oxosteroid 5β-reductase, which catalyzes the reduction of molecules with a 3-oxo-4-ene structure in the presence of nicotinamide adenine dinucleotide phosphate (NADPH) as a hydride donor [16–18]. AKR1D1 is required for cholesterol metabolism into bile acids. In the bile acid synthesis pathway, AKR1D1 catalyzes a reaction downstream of the reaction mediated by CYP7A1. It reduces the double bond in the A ring of the bile acid intermediates to eventually produce primary bile acids [19–21]. Consistent with its vital role in bile acid synthesis, deficiency in AKR1D1 expression or activity has been directly linked to severe cholestatic liver disease in infancy, with characteristic appearance of unsaturated 3-oxo-4-ene-bile acids [22–27]. In addition to its critical role in bile acid synthesis, AKR1D1 is also involved in steroid hormone metabolism and clearance [28, 29]. Most of the steroid hormones including testosterones, progesterones, mineralocorticoids and glucocorticoids carry the 3-oxo-4-ene structure. Since 5β-reduction is a common transformation and major deactivation pathway for steroid hormones, and AKR1D1 is the only enzyme in humans capable of catalyzing a 5β-reduction in those steroids, AKR1D1 plays a critical role in regulating and maintaining the homeostasis of steroid hormones [19, 22, 28, 29]. Taken together, AKR1D1 is sitting at the interface between the two important biological pathways, bile acid synthesis and steroid hormone metabolism, both of which have a broad spectrum of impacts on metabolisms and energy expenditure. However, our current understanding on the regulation of AKR1D1 expression under physiological and pathological conditions is very limited.

In this study, we investigated the regulatory effects of bile acids on AKR1D1 expression. Chenodeoxycholic acid (CDCA) and cholic acid (CA) are the two primary bile acids in human. We found that CDCA markedly repressed AKR1D1 expression *in vitro* in human hepatoma HepG2 cells and *in vivo* in mice. In contrast, CA significantly upregulated AKR1D1 expression in HepG2 cells and in mice. Further mechanistic studies revealed that the FXR signaling pathway was not involved in regulating AKR1D1 by bile acids. Instead, CDCA and CA regulated AKR1D1 expression through the mitogen-activated protein kinases/c-Jun N-terminal kinases (MAPK/JNK) signaling pathway. Inhibition of the MAPK/JNK pathway effectively abolished CDCA and CA-mediated regulatory effects on AKR1D1. It was thus concluded that AKR1D1 expression was differentially regulated by CDCA and CA through modulating the MAPK/JNK signaling pathway.

## Materials and Methods

### Chemicals and reagents

CDCA and CA were purchased from Sigma-Aldrich. FXR agonist GW4064 was obtained from Tocris Biosciences. Cell culture reagents Dulbecco’s modified Eagle’s medium (DMEM), fetal bovine serum (FBS), TaqMan master-mix and probes were purchased from Life Technologies. RNA Bee for RNA isolation, dimethyl sulfoxide (DMSO), and propanediol were obtained from Fisher Scientific. Primary and secondary antibodies for Western blotting were purchased from Santa Cruz Biotechnologies. All Western blotting gels, buffers, and markers were purchased through BioRad Laboratories. Complementary DNA synthesis kit was purchased through Promega. Protease inhibitor, Halt, and bovine serum albumin quantification reagents were purchased from Thermo Scientific.

### Treatment of HepG2 cells

Human hepatoma HepG2 cells (ATCC, HB-8065) were seeded in 12-well plates at a cell density of 4.0 x 10^5^ in 1.0 mL DMEM supplemented with 10% (v/v) FBS, 1% (v/v) penicillin/streptomycin and 1% (v/v) non-essential amino acids (NEAA) and cultured in a 5% CO_2_ incubator overnight at 37°C before treatment. After overnight incubation, cells were treated with 25 μM CDCA, 25 μM CA, 1 μM GW4064 or vehicle DMSO for 30 hours. Total mRNA and protein lysates were prepared for real-time PCR and Western blot. HepG2 cells seeded in 12-well plates were transfected with FXRα1 or FXRα2 expression plasmid (1μg/well) [30], followed by treatment of the transfected cells with FXR agonist GW4064 for 30 h. Total cellular RNAs were isolated for real-time PCR analyses. HepG2 cells were treated with CDCA (25μM), CDCA+MAPK/JNK inhibitor SP600125 (1μM), CDCA+MAPK/ERK1/2 inhibitor PD98059 (5μM), CA (50μM), CA+SP600125, CA+PD98059, or vehicle for 30 hours, followed by preparation of total RNA for real-time PCR and cell lysates for Western blot analysis.

### Treatment of mice

Twenty four male and female CD-1 mice (*mus musculus*) were bred in-house and randomly divided into 4 groups with 6 mice per group at ages of 6–8 weeks. The mice had free access to food and water and were on a 12-hour dark/light cycle. The mice were treated with CDCA (5mg/kg), CA (5mg/kg), GW4064 (1mg/kg) or vehicle propanediol through intraperitoneal injection twice a day for 3 days. Twelve hours after the last injection, mice were euthanized by CO_2_ euthanasia method and liver tissues were harvested and processed for mRNA and protein analyses. In addition, a group of wild type (Wt) and FXR-knockout mice (FXR-/-) were euthanized with CO_2_ euthanasia method and liver tissues were collected for gene expression analysis. The Institutional Animal Care and Use Committee (IACUC) at the University of Rhode Island approved all animal studies with a protocol number of AN09-02-008.

### Quantitative real-time PCR

Total RNA isolation from HepG2 cells or mouse liver tissues and subsequent TaqMan real-time PCR were carried out as described [31, 32]. Transcript levels of AKR1D1, CYP7A1, CYP8B1 and BSEP were quantified by real-time PCR with gene-specific probes. The expression levels of target mRNA were normalized against the mRNA levels of glyceraldehyde-3-phosphate dehydrogenase (GAPDH).

### Western blotting

Cell lysates were made from HepG2 or mouse liver tissues as described [33, 34]. Thirty micrograms of total protein were loaded into each well. Membranes were blotted with antibodies against human or mouse AKR1D1, CYP7A1, CYP8B1 or BSEP (Santa Cruz Biotechnologies). The same membranes were stripped and re-blotted with antibodies against GAPDH (Sigma-Aldrich). Quantifications of AKR1D1, CYP7A1, CYP8B1 and BSEP protein levels were performed with all the samples in each individual groups or treatments. Representative images of Western blot from each individual groups or treatments were presented. The expression levels of GAPDH were used to normalize the expression of target genes [35].

### Signal Transduction 45-Pathway Reporter Array

The Cignal 45-Pathway Reporter Array was purchased from Qiagen (Cat#: CCA-901L). The array assays were essentially carried out according to the protocol recommended by the manufacture. Reverse transfection was performed using HepG2 cells (8x10^4^ cells/well in 98-well plates). Sixteen hours post-transfection, transfected cells were treated with CDCA (25 μM), CA (25 μM) or vehicle DMSO for 30 hours, followed by detection of luciferase activities by the Dual Luciferase Assay [36].

### Statistical analysis

Student’s t-test was applied to pair-wise comparison for normally distributed data. One-way ANOVA was applied to analyze data with multiple groups, followed by Tukey post-hoc test for multiple comparisons. Non-parametric Mann-Whitney test was used for pair-wise comparison for non-normally distributed data. A p value of 0.05 or less was considered statistically significant.

## Results

### Bile acid CDCA markedly repressed AKR1D1 expression in human hepatoma HepG2 cells

**Fig 1:**
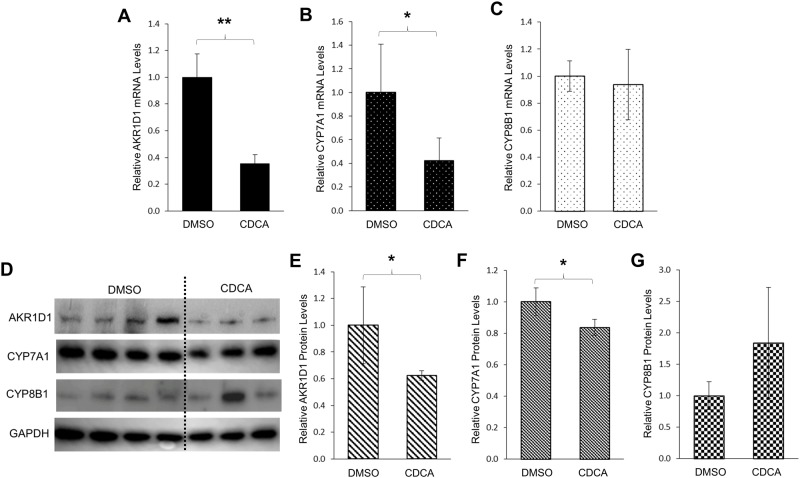
CDCA markedly repressed AKR1D1 expression in human hepatoma HepG2 cells. HepG2 cells were treated with CDCA (25μM) or vehicle DMSO (0.1%) for 30h, followed by detection of the mRNA levels of (A) AKR1D1, (B) CYP7A1 and (C) CYP8B1 by real-time PCR, and (D) protein levels of AKR1D1, CYP7A1 and CYP8B1 by Western blotting. (E) quantification of AKR1D1, (F) CYP7A1, and (G) CYP8B1 protein levels in (D). The data are presented as mean ± SD of at least three separate experiments or treatments. The Student’s t-test was applied to pair-wise comparison. *p<0.05, **p<0.01.

CDCA is one of the major primary bile acids in human. AKR1D1 is an enzyme involved in both bile acid synthesis and steroid hormone metabolism. To investigate whether AKR1D1 expression is regulated by its ultimate product CDCA, the effects of CDCA treatment on AKR1D1 expression were determined in human hepatoma HepG2 cells. As shown in Fig 1A, AKR1D1 mRNA expression levels were markedly decreased by 65% in cells treated with CDCA in comparison with the levels in cells treated with vehicle. It is well established that CDCA down-regulates CYP7A1 through a negative feedback loop [8, 9]. Indeed, CDCA treatment reduced CYP7A1 mRNA levels by 58% (Fig 1B). However, the expression levels of CYP8B1 mRNA were not significantly changed (Fig 1C). Consistent with the altered mRNA levels, CDCA treatment significantly reduced the protein levels of AKR1D1 (Fig 1D and 1E) by 40%. As expected, CYP7A1 protein levels were also significantly decreased (Fig 1D and 1F). On the other hand, CYP8B1 protein levels were slightly increased without reaching a statistical significance (Fig 1D and 1G). The data thus demonstrated that CDCA repressed AKR1D1 expression in human HepG2 cells.

**Fig 2:**
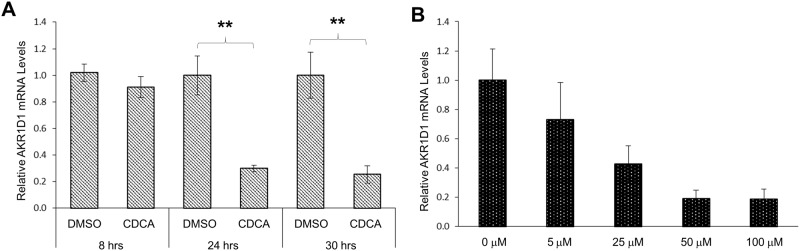
CDCA repressed AKR1D1 expression in time and dose-dependent manners in HepG2 cells. (A) HepG2 cells were treated with either CDCA (25 μM) or vehicle DMSO for 8, 24 or 30 hrs, followed by detection of AKR1D1 mRNA with real-time PCR. (B) HepG2 cells were treated with various concentrations of CDCA (0, 5, 25, 50, 100 μM) for 30 hrs, followed by detection of AKR1D1 mRNA expression with real-time PCR. **p<0.01 with the Student’s t-test for pair-wise comparison.

A time course study revealed that no significant repression of AKR1D1 expression by CDCA was detected 8 hours (hrs) post-treatment. However, marked repressions of AKR1D1 (70% and 75% reduction) were detected 24 and 30 hrs post-treatment of CDCA (Fig 2A). A dose-response study showed that CDCA dose-dependently repressed AKR1D1 expression (Fig 2B). AKR1D1 expression levels were decreased by 27%, 57%, 81% and 81% in cells treated with 5, 25, 50 and 100 μM CDCA, respectively. Taken together, the results demonstrated that AKR1D1 expression was repressed by CDCA in time and dose-dependent manners.

### Akr1d1 expression was severely repressed by CDCA *in vivo* in mice

**Fig 3:**
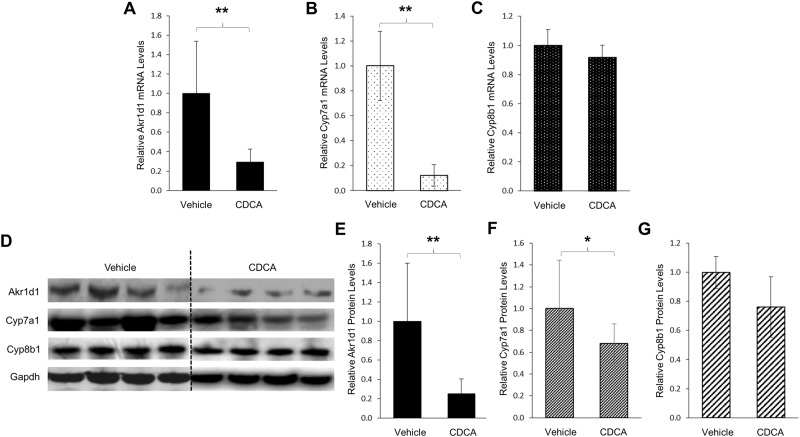
Akr1d1 expression was severely repressed by CDCA *in vivo* in mice. Two groups of mice (n = 6/group) were treated with CDCA (5mg/kg) or vehicle propanediol through intraperitoneal injection twice a day for 3 days. Twelve hours after the last injection, mice were euthanized and liver tissues were harvested and processed for mRNA and protein analyses. (A) the effects of CDCA treatment on Akr1d1, (B) Cyp7a1 and (C) Cyp8b1 mRNA levels detected by real-time PCR. (D) the effects of CDCA treatment on Akr1d1, Cyp7a1 and Cyp8b1 protein expression detected by Western blotting, and (E) quantification of Akr1d1, (F) Cyp7a1 and (G) Cyp8b1 protein levels in (). The data are presented as mean ± SD of the groups. The Student’s t-test was applied to pair-wise comparison. *p<0.05, **p<0.01.

To determine whether CDCA-mediated regulation of AKR1D1 in HepG2 cells is conserved *in vivo* in mice, we carried out a mouse study to determine the effects of CDCA on Akr1d1 expression. As shown in Fig 3A, Akr1d1 mRNA levels were severely repressed by CDCA. Compared with vehicle control, Akr1d1 mRNA levels were decreased by 70%. As expected, CDCA treatment markedly decreased Cyp7a1 mRNA levels by 88% (Fig 3B). However, Cyp8b1 mRNA levels were minimally changed by CDCA treatment (Fig 3C). Consistently, Akr1d1 protein levels were decreased by 75% in mice treated with CDCA when compared with vehicle-treated mice (Fig 3D and 3E). Similarly, the Cyp7a1 protein levels were significantly decreased by CDCA treatment (Fig 3D and 3F). On the other hand, no significant changes in CYP8B1 protein levels were detected (Fig 3D and 3G). Taken together, the data demonstrated that consistent with the results from human HepG2 cells, CDCA down-regulated AKR1D1 expression *in vivo* in mice. It was thus concluded that similar to CYP7A1, AKR1D1 was regulated by CDCA in a negative feedback manner.

### Bile acid CA significantly upregulated AKR1D1 expression in human HepG2 cells

**Fig 4:**
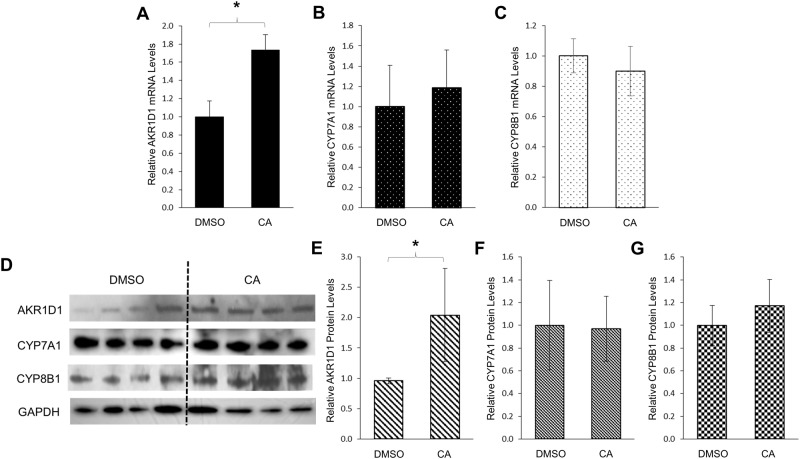
CA significantly upregulated AKR1D1 expression in human HepG2 cells. HepG2 cells were treated with CA (25μM) or vehicle DMSO (0.1%) for 30h, followed by detection of the mRNA levels of (A) AKR1D1, (B) CYP7A1 and (C) CYP8B1 by real-time PCR, and the protein levels of (D) AKR1D1, CYP7A1 and CYP8B1 detected by Western blotting. (E) quantification of AKR1D1, (F) CPY7A1 and (G) CYP8B1 protein levels in (D). The data are presented as mean ± SD of at least three separate experiments or treatments. The Student’s t-test was applied to pair-wise comparison. *p<0.05.

CA is the other primary bile acid in human. To determine whether CA acts similarly as CDCA in modulating AKR1D1 expression, the effects of CA treatment on AKR1D1 expression was investigated in HepG2 cells. As shown in Fig 4A, in contrast to CDCA, CA treatment significantly increased AKR1D1 mRNA levels by 74% when compared with vehicle. On the other hand, the expression levels of CYP7A1 and CYP8B1 mRNA were not significantly altered by CA treatment (Fig 4B and 4C). In line with mRNA levels, AKR1D1 protein levels were increased by 1.1 fold in cells treated with CA (Fig 4D and 4E) while CYP7A1 and CYP8B1 protein levels were minimally changed. (Fig 4D, 4F and 4G).

**Fig 5:**
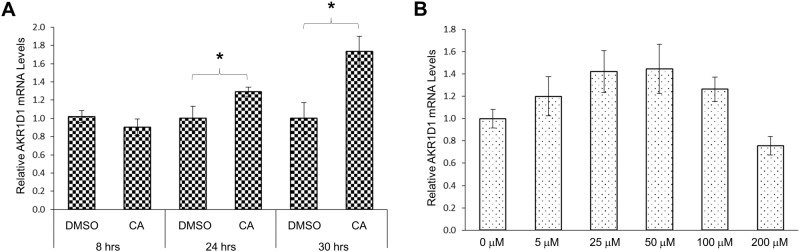
CA modulated AKR1D1 expression in time and dose-dependent manners in HepG2 cells. (A) HepG2 cells were treated with either CA (25 μM) or vehicle DMSO for 8, 24 or 30 hrs, followed by detection of AKR1D1 mRNA with real-time PCR. (B) HepG2 cells were treated with various concentrations of CA (0, 5, 25, 50, 100, 200 μM) for 30 hrs, followed by detection of AKR1D1 mRNA expression with real-time PCR. *p<0.05 with the Student’s t-test for pair-wise comparison.

A time course study showed that minimal changes in AKR1D1 expression were detected in cells treated with CA for 8 hrs. A significant 30% increase in AKR1D1 expression was detected in cells treated with CA for 24 hrs with additional increases up to 74% being detected in cells treated with CA for 30 hrs (Fig 5A). A dosing study revealed an atypical response in AKR1D1 expression following treatments with various concentrations of CA (Fig 5B). From the dose range of 0 to 50 μM, AKR1D1 expression levels were gradually increased as CA concentrations increased. However, from the dose range of 50 to 200 μM, AKR1D1 expression levels were gradually decreased as CA concentrations increased (Fig 5B). Therefore, CA-mediated modulating effects on AKR1D1 expression were dose-dependent and binary.

### Akr1d1 expression was induced by CA *in vivo* in mice

**Fig 6:**
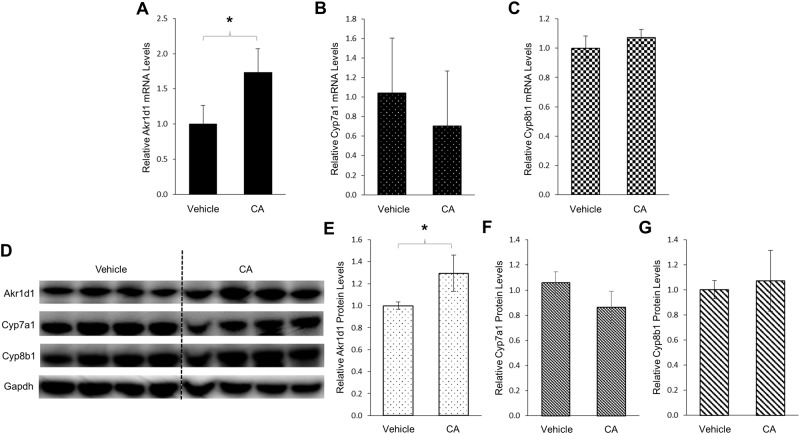
Akr1d1 expression was induced by CA *in vivo* in mice. Two groups of mice (n = 6/group) were treated with CA (5mg/kg) or vehicle propanediol through intraperitoneal injection twice a day for 3 days. Twelve hours after the last injection, mice were euthanized and liver tissues were harvested and processed for mRNA and protein analyses. (A) the effects of CA treatment on Akr1d1, (B) Cyp7a1 and (C) Cyp8b1 mRNA levels detected by real-time PCR. (D) the effects of CA treatment on Akr1d1, Cyp7a1 and Cyp8b1 protein expression detected by Western blotting, and (E) quantification of Akr1d1, (F) Cyp7a1 and (G) Cyp8b1 protein levels in (D). The data are presented as mean ± SD of the groups. The Student’s t-test was applied to pair-wise comparison. *p<0.05.

To confirm the findings that AKR1D1 expression was upregulated by CA in HepG2 cells, we performed a mouse study to determine the effects of CA on Akr1d1 expression *in vivo*. As shown in Fig 6A, CA treatment significantly increased the expression levels of Akr1d1 mRNA by 73%. On the other hand, Cyp7a1 expression was slightly decreased by CA treatment without reaching statistical significance (Fig 6B). Minimal changes in Cyp8b1 mRNA levels were detected in mice treated with CA (Fig 6C). Consistent with the results of mRNA levels, Akr1d1 protein levels were significantly increased by 36% following CA treatment (Fig 6D and 6E) while a slight decrease in Cyp7a1 and minimal change in Cyp8b1 protein levels were detected (Fig 6F and 6G). The data thus demonstrated that in contrast to CDCA, CA induced AKR1D1 expression *in vitro* in human HepG2 cells and *in vivo* in mice. Taken together, the results revealed that bile acids CDCA and CA differentially down- and upregulated AKR1D1 expression.

### FXR signaling was not involved in regulating AKR1D1

**Fig 7:**
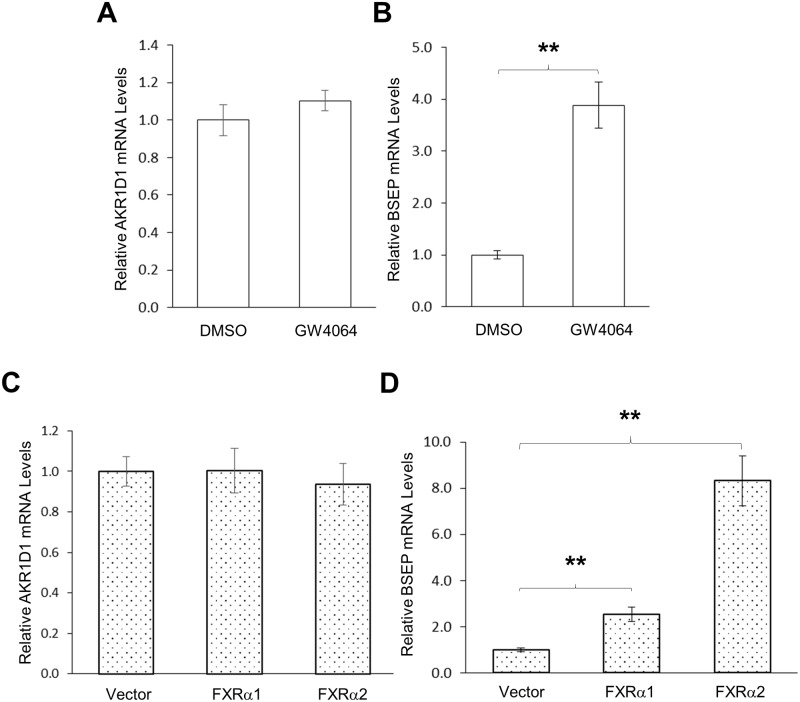
FXR signaling was not involved in regulating AKR1D1 by bile acids in HepG2 cells. (A) HepG2 cells were treated with FXR agonist GW4064 (1μM) or vehicle DMSO (0.1%) for 30h, followed by detection of AKR1D1 and BSEP mRNA levels by real-time PCR. (B) HepG2 cells were transfected with FXRα1, FXRα2 or vector, followed by treatment with GW4064 (1μM) for 30h. The expression levels of AKR1D1 and BSEP were detected by real-time PCR. The Student’s t-test was applied to pair-wise comparison. One-way ANOVA was applied to analyze data with multiple groups, followed by Tukey post-hoc test for multiple comparisons. ** p<0.01.

FXR is the master regulator of bile acid homeostasis [1, 2], and bile acids including CDCA and CA are endogenous ligands for FXR with different agonistic activities. To determine whether FXR is involved in CDCA and CA-mediated regulation of AKR1D1, we performed a series of experiments. First, we determined whether activation of FXR by a synthetic agonist GW4064 has any effects on AKR1D1 expression *in vitro* in HepG2 cells. As shown in Fig 7A, activation of FXR by GW4064 had minimal effects on AKR1D1 expression while FXR target gene BSEP was markedly induced by GW4064 in HepG2 cells (Fig 7B). Next, we evaluated the effects of FXR over-expression on AKR1D1 expression. As shown in Fig 7C, over-expression of FXRα1 or FXRα2 had minimal effects on AKR1D1 expression in HepG2 cells. On the other hand, as expected, over-expression of FXRα1 or FXRα2 significantly upregulated BSEP expression (Fig 7D). The results indicated that FXR signaling was not involved in regulating AKR1D1 in human HepG2 cells.

**Fig 8:**
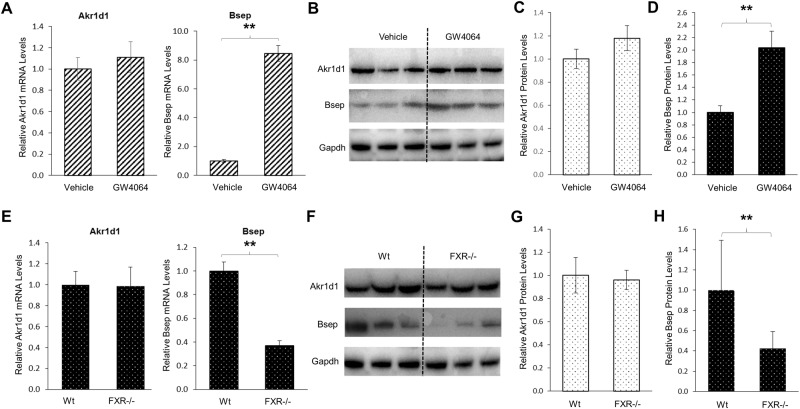
FXR signaling was not involved in regulating AKR1D1 by bile acids *in vivo* in mice. (A) two groups of mice (n = 6/group) were treated with GW4064 (5mg/kg) or vehicle propanediol through intraperitoneal injection twice a day for 3 days. The expression levels of Akr1d1 and Bsep were quantified by real-time PCR and (B) Western blot. (C) quantification of Akr1d1 and (D) Bsep protein levels in (B). (E) the expression levels of Akr1d1 and Bsep in wt and FXR-knockout (FXR-/-) mice were determined by real-time PCR and (F) Western blot. (G) quantification of Akr1d1 and (H) Bsep protein levels in (F). The data are presented as mean ± SD of the groups of mice. The Student’s t-test was applied to pair-wise comparison. ** p<0.01.

To confirm the findings, we carried out a mouse study to determine the effects of FXR activation on Akr1d1 expression *in vivo*. Consistent with the results from HepG2 cells, activation of FXR by its agonist GW4064 *in vivo* in mice did not significantly alter Akr1d1 expression at both mRNA and protein levels (Fig 8A, 8B and 8C) while it did markedly induced FXR target Bsep expression at both mRNA and protein levels (Fig 8A, 8B and 8D). Finally, we compared Akr1d1 expression levels in wt and FXR knockout (FXR-/-) mice. No significant differences in the expression levels of Akr1d1 at both mRNA and protein levels were detected between the wt and FXR-/- mice (Fig 8E, 8F and 8G). On the other hand, as expected, Bsep expressions at both mRNA and protein levels were significantly reduced in FXR-/- mice (Fig 8E, 8F and 8H). The consistent data from those series of *in vitro* and *in vivo* experiments concluded that FXR signaling was not involved in regulating AKR1D1 expression, thus excluded the possibility that CDCA and CA differentially regulated AKR1D1 through the FXR signaling pathway.

### CDCA and CA differentially modulated the MAPK/JNK signaling pathway

**Fig 9:**
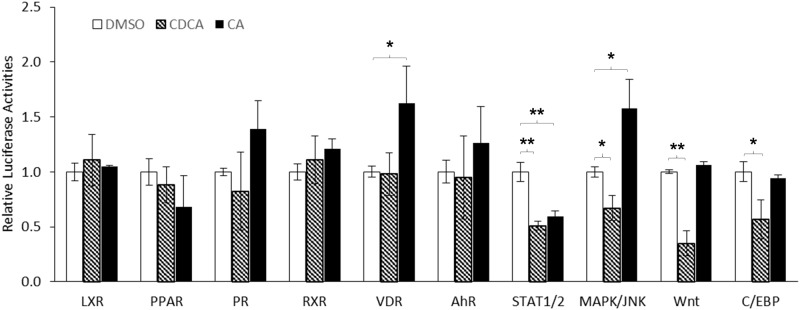
CDCA and CA differentially modulated the MAPK/JNK signaling pathway. HepG2 cells were reversely transfected with 45 signaling pathway element reporter plasmids, followed by treatment of transfected cells with CDCA (25μM), CA (25μM) or vehicle DMSO (0.1%) for 30 h. The luciferase activities were detected with the Dual Luciferase Assays. The data are presented as mean ± SD of at least three separate experiments. One-way ANOVA was applied to analyze data with multiple groups, followed by Tukey post-hoc test for multiple comparisons. * p<0.05 and ** p<0.01.

To identify potential signaling pathways through which CDCA and CA differentially regulate AKR1D1 expression, we screened 45 signaling pathways using Signal Transduction 45-Pathway Reporter Array. CDCA and CA did not show significant effects on most of the signaling pathways (data not shown). A set of representative data including the signaling pathways altered by CDCA, CA or both were presented in Fig 9. CDCA treatment had no significant effects on various nuclear receptor pathways including liver x receptor (LXR), peroxisome proliferator-activated receptor (PPAR), progesterone receptor (PR), retinoid X receptor (RXR), vitamin D receptor (VDR) and aryl hydrocarbon receptor (AhR). However, CDCA significantly down-regulated the following signaling pathways including the signal transducer and activator of transcription 1 and 2 (STAT1/2), mitogen-activated protein kinases/c-Jun N-terminal kinases (MAPK/JNK), Wnt and CCAAT-enhancer-binding proteins (C/EBP). For CA treatment, minimal effects on the following signaling pathways were detected including LXR, PPAR, PR, RXR, AhR, Wnt and C/EBP. However, the VDR and MAPK/JNK signaling were significantly elevated by CA treatment. On the other hand, CA down-regulated STAT1/2 signaling pathways. Taken together, among those signaling pathways, CDCA and CA differentially down- and upregulated MAPK/JNK signaling pathway, which exhibits the similar regulatory pattern of AKR1D1 following CDCA and CA treatments. Therefore, the MAPK/JNK pathway was the candidate pathway through which CDCA and CA differentially regulated AKR1D1.

### Inhibition of MAPK/JNK signaling pathway abolished CDCA and CA-mediated regulatory effects on AKR1D1

**Fig 10:**
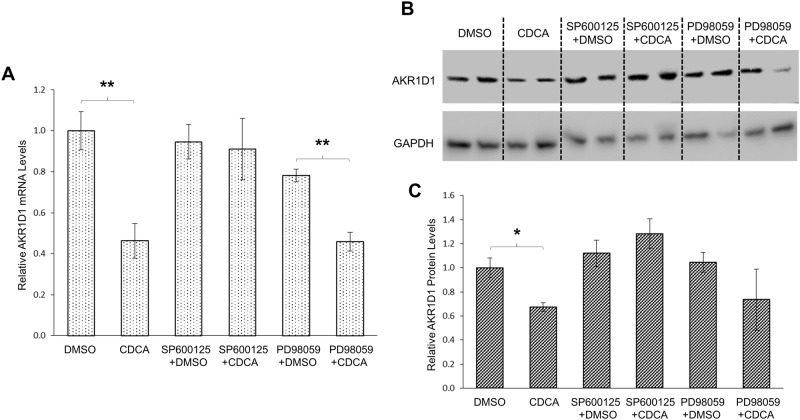
Inhibition of MAPK/JNK signaling pathway abolished CDCA-mediated regulation of AKR1D1. (A) HepG2 cells were treated with CDCA (25μM) in the absence or presence of MAPK/JNK inhibitor SP600125 (1μM), MAPK/ERK1/2 inhibitor PD98059 (5μM) or vehicle for 30 hrs, followed by detection of AKR1D1 mRNA by real-time PCR and (B) AKR1D1 protein by Western blot. (C) quantification of AKR1D1 protein levels in (B). One-way ANOVA was applied to analyze the data, followed by Tukey post-hoc test for multiple comparisons. * p<0.05 and ** p<0.01.

**Fig 11:**
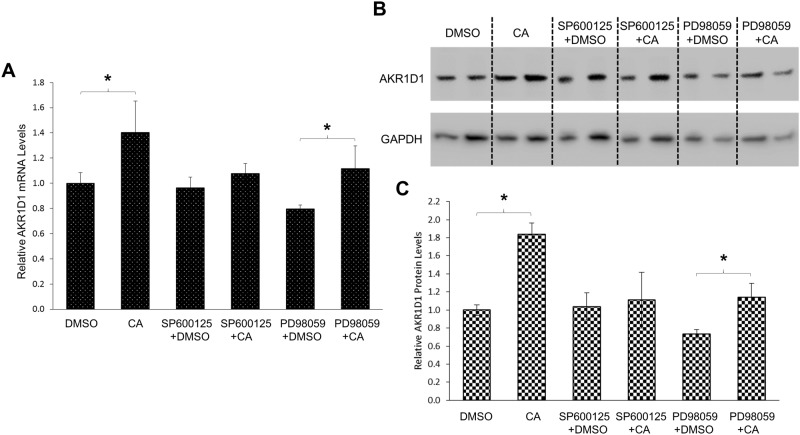
Inhibition of MAPK/JNK signaling pathway abolished CA-mediated regulation of AKR1D1. (A) HepG2 cells were treated with CA (50μM) in the absence or presence of MAPK/JNK inhibitor SP600125 (1μM), MAPK/ERK1/2 inhibitor PD98059 (5μM) or vehicle for 30 hrs, followed by detection of AKR1D1 mRNA by real-time PCR and (B) AKR1D1 protein by Western blot. (C) quantification of AKR1D1 protein levels in (B). One-way ANOVA was applied to analyze the data, followed by Tukey post-hoc test for multiple comparisons. * p<0.05.

To determine whether CDCA and CA regulated AKR1D1 expression through the MAPK/JNK signaling pathway, the effects of MAPK/JNK inhibition on CDCA or CA-mediated regulation of AKR1D1 were evaluated in HepG2 cells. The AKR1D1 expression levels were detected in the absence or presence of a selective MAPK/JNK inhibitor, SP600125. A selective MAPK/extracellular signal-regulated kinases 1/2 (ERK1/2) inhibitor, PD98059, was included in the experiments as a negative control. As shown in Fig 10A, CDCA significantly decreased AKR1D1 mRNA expression by 54% while SP600125 had minimal effects. However, such CDCA-mediated decrease in AKR1D1 mRNA expression was completely abolished in the presence of MAPK/JNK inhibitor SP600125. On the other hand, the ability of CDCA to repress AKR1D1 mRNA expression remained intact in the presence of MAPK/ERK1/2 inhibitor PD98059. AKR1D1 mRNA expression was slightly decreased by PD98059 alone and was further significantly reduced by CDCA. Consistent with the results of AKR1D1 mRNA levels, AKR1D1 protein expression was significantly repressed by CDCA (Fig 10B and 10C). However, such CDCA-mediated repression of AKR1D1 protein expression was totally abolished in the presence of MAPK/JNK inhibitor SP600125. In contrast, AKR1D1 protein levels were slightly increased in cells treated with SP600125 and CDCA. On the other hand, CDCA treatment resulted in decreased AKR1D1 protein expression by 27% in the presence of PD98059. The consistent results of CDCA’s effects on both AKR1D1 mRNA and protein expression thus established that MAPK/JNK signaling pathway was involved in CDCA-mediated downregulation of AKR1D1 expression. Similar treatments were applied to determine whether MAPK/JNK signaling is involved in CA-mediated regulation of AKR1D1. As shown in Fig 11A, CA treatment increased AKR1D1 mRNA expression by 40%. However such CA-mediated induction of AKR1D1 mRNA expression was completely abolished by MAPK/JNK inhibitor SP600125 but not MAPK/ERK1/2 inhibitor PD98059. Consistent modulating effects of CA on AKR1D1 protein levels were detected (Fig 11B and 11C) in the absence and presence of MAPK/JNK inhibitor. CA treatment induced AKR1D1 protein expression by 84%. However, such induction was blunted in the presence of SP600125. Treatment with PD98059 reduced AKR1D1 expression. However, addition of CA significantly elevated AKR1D1 protein levels by 54%. The results thus demonstrated that MAPK/JNK signaling was involved in CA-mediated upregulation of AKR1D1. Taken together, the data revealed that MAPK/JNK signaling was involved in CDCA and CA-mediated differential regulation of AKR1D1 expression. It was thus concluded that CDCA and CA differentially regulated AKR1D1 expression through modulating the MAPK/JNK signaling pathway.

## Discussion

**Fig 12:**
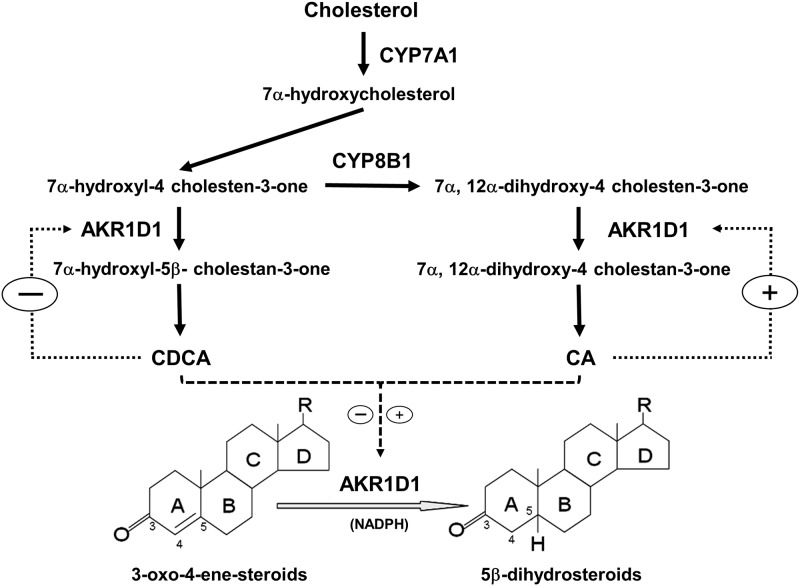
Functions and regulation of AKR1D1 in bile acid synthesis and steroid hormone metabolism. In the bile acid synthesis pathway, bile acid intermediate 7α-hydroxy-4-cholesten-3-one can take one of the two routes in subsequent steps. If the intermediate is acted upon by AKR1D1, the ultimate product is CDCA. If the intermediate is acted upon by CYP8B1, followed by AKR1D1, the ultimate product is CA. In this study, we demonstrated that CDCA and CA regulated AKR1D1 through a negative and positive feedback mechanism, respectively. In addition to bile acid synthesis, AKR1D1 is also involved in steroid hormone metabolism. 5β-reduction by AKR1D1 is a common transformation and major deactivation pathway for many steroid hormones. Therefore, AKR1D1 plays a critical role in regulating and maintaining the homeostasis of steroid hormones. Thus bile acids crosstalk with steroid hormone signaling pathways through modulating AKR1D1 expression. Plus (+) and minus (-) indicated positive and negative feedback regulation, respectively.

In the bile acid synthesis pathway, AKR1D1 catalyzes the 5β-reduction of bile acid intermediates to eventually produce the primary bile acids CDCA and CA. It is well established that CYP7A1-mediated 7α-hydroxylation is the rate-limiting step in bile acid synthesis and bile acid homeostasis is maintained by regulating CYP7A1 expression through several negative feedback mechanisms [8–10]. In this study, we found that AKR1D1 was differentially regulated by the two primary bile acids. Similar to the effects on CYP7A1, CDCA markedly repressed AKR1D1 expression (Figs 1 and 3). On the other hand, CA induced AKR1D1 expression while CYP7A1 expression was minimally affected by CA (Figs 4 and 6). Our new findings indicate that bile acid synthesis is regulated at multiple steps in the bile acid synthesis pathway. In addition to CYP7A1, AKR1D1-mediated 5β-reduction of bile acid intermediates is also regulated by bile acids. CDCA-mediated repression of AKR1D1 expression represents a negative feedback mechanism to restrain bile acid production while CA-mediated upregulation of AKR1D1 represents a feed-forward regulation loop to promote bile acid production (Fig 12). The data thus suggest that both bile acid concentration and composition have an impact on controlling bile acid synthesis.

Various bile acid species exhibited diverse or even opposite biochemical and physiological properties. For example, CA promotes while CDCA inhibits intestinal cholesterol adsorption [37–40]. Alterations in bile acid compositions are associated with various pathological conditions [41–44]. It is therefore critical to maintain a normal bile acid composition. Bile acid composition is mainly determined by the relative concentrations of the two primary bile acids CDCA and CA or the ratio of CA/CDCA. CYP8B1 is the determining factor for the production of CA and its expression levels dictate the bile acid composition or the ratio of CA/CDCA [11, 45]. However, as shown in Fig 8, in the bile acid synthesis pathway, bile acid intermediate 7α-hydroxy-4-cholesten-3-one can take one of the two routes in subsequent steps. If the intermediate is acted upon by AKR1D1, the ultimate product is CDCA. If the intermediate is acted upon by CYP8B1, followed by AKR1D1, the ultimate product is CA (Fig 12). Therefore, the relative CA and CDCA production or the CA/CDCA ratio, is determined by both CYP8B1 and AKR1D1. It is speculated that AKR1D1 works together with CYP8B1 to coordinately maintain the relative production of CDCA and CA under physiological condition. Repression of AKR1D1 by CDCA is a typical negative feedback mechanism to control the production of CDCA. On the other hand, upregulation of AKR1D1 by CA represents a feed-forward regulation of CA production (Fig 12).

In addition to bile acid synthesis, AKR1D1 is involved in steroid hormone metabolism [28, 29]. 5β-reduction by AKR1D1 is a common transformation and major deactivation pathway for many steroid hormones. Therefore, AKR1D1 plays a critical role in regulating and maintaining the homeostasis of steroid hormones [19, 22, 28, 29]. In this investigation, we revealed that AKR1D1 expression was regulated by bile acids. It was thus speculated that bile acids may interplay with steroid hormone signaling pathways through modulating AKR1D1 expression (Fig 12). Indeed, bile acids were reported to modulate glucocorticoid metabolism and had an impact on the hypothalamic-pituitary-adrenal axis [46]. On the other hand, steroid hormones such as glucocorticoids are capable of directly or indirectly regulating bile acid homeostasis [47]. It remains to be determined how bile acid and steroid hormone signaling pathways crosstalk each other, especially through AKR1D1.

It is well established that bile acids down-regulate CYP7A1 expression through activating FXR [8–11]. Activation of FXR by bile acids induce the expression of small heterodimer partner (SHP) in the liver and fibroblast growth factor 19 (FGF19) in the intestine, which in turn repress CYP7A1 expression. In the current study, it was surprisingly found that FXR signaling pathway was not involved in bile acids-mediated regulation of AKR1D1. Activation of FXR by synthetic FXR agonist GW4064 *in vitro* and *in vivo* or knockout of FXR *in vivo* had minimal effects on AKR1D1 expression. Instead, we discovered that bile acids-mediated regulation of AKR1D1 involved the MAPK/JNK signaling pathway. Blockage of the MAPK/JNK signaling pathway totally abolished CDCA and CA-mediated regulatory effects on AKR1D1. The connection between bile acids and MAPK signaling pathways have long been recognized [48–53]. Previous studies showed that MAPK/JNK and MAPK/ERK1/2 signaling pathways also contribute to the down-regulation of CYP7A1 by bile acids in addition to the FXR signaling pathway [48, 49, 54, 55]. The MAPK signal transduction pathway is one of the most important regulatory mechanisms in eukaryotic cells that transduce environmental and developmental signals into adaptive and programmed responses including cell proliferation, differentiation and death, and inflammation. The JNKs were initially identified as the stress-activated protein kinases and are mainly activated in response to stress and proinflammatory cytokines [56]. Bile acids-mediated regulation of AKR1D1 and CYP7A1 through MAPK/JNK signaling pathways may represent a mechanism for the cells to cope with cellular stress and inflammation while bile acids-mediated feedback repression of CYP7A1 through FXR signaling pathway represents the main mechanism for hepatocytes to maintain bile acid homeostasis.

It was well documented that different bile acid species exhibit various regulatory effects on their target genes through modulating the MAPK signaling pathways. CA was identified as the most potent bile acid to activate MAPK/JNK while CDCA exhibited a much less potency in rat primary hepatocytes [55]. Deoxycholic acid (DCA) was found to be the most potent activator of MAPK/ERK signaling when compared with other bile acids [57]. DCA and UDCA exhibited opposite effects on colon cancer progression by differentially modulating MAPK pathways [58]. On the other hand, the same bile acids exhibited different or even opposite effects on various target genes by modulating the MAPK pathways. For examples, CDCA down-regulated CYP7A1 and acetyl-coenzyme A carboxylase-α (ACCα) while upregulated early growth response factor-1 (EGR-1) and low-density lipoprotein receptor (LDLR) through differentially modulating the MAPK signaling pathways [48–55]. In this investigation, CDCA and CA regulated AKR1D1 through the MAPK/JNK pathway with opposite effects. The underlying mechanisms for such differential effects of CDCA and CA on AKR1D1 expression remain to be determined. One possible explanation is that CDCA and CA act upon different upstream stress or inflammation-related signals in the MAPK/JNK cascade. Supporting such speculation is our data that CDCA-mediated down-regulation of AKR1D1 in HepG2 cells was consistently detected regardless cell sources and passage history. In contrast, CA-mediated upregulation of AKR1D1 was cell status-dependent (the source and passage history of the HepG2 cells). In addition, CDCA exhibited a linear (Fig 2B) while CA showed a binary dose-response in modulating AKR1D1 expression in HepG2 cells (Fig 5B). Taken together, AKR1D1 expression was differentially regulated by CDCA and CA, and such feedback regulation was mediated through the MAPK/JNK signaling pathway.
